# Influence of d-Electron Divalent Metal Ions in Complex Formation with L-Tartaric and L-Malic Acids

**DOI:** 10.3390/molecules26175290

**Published:** 2021-08-31

**Authors:** Michał Zabiszak, Justyna Frymark, Martyna Nowak, Jakub Grajewski, Klaudia Stachowiak, Małgorzata T. Kaczmarek, Renata Jastrząb

**Affiliations:** Faculty of Chemistry, Adami Mickiewicz University in Poznan, Uniwersytetu Poznańskiego 8, 61-614 Poznan, Poland; jusfry@st.amu.edu.pl (J.F.); martynan@amu.edu.pl (M.N.); jakub.grajewski@amu.edu.pl (J.G.); klasta5@st.amu.edu.pl (K.S.); gosiat@amu.edu.pl (M.T.K.); renatad@amu.edu.pl (R.J.)

**Keywords:** d-electron complexes, tartaric acid, malic acid, potentiometric measurements, spectroscopic studies

## Abstract

Binary complexes of α-hydroxy acids (L-Tartaric acid and L-Malic acid) with d-electron metal ions (copper, cobalt, nickel) were investigated. Potentiometric measurements have been performed in aqueous solution with computer analysis of the data for determination of the stability constants of complexes formed in the studied systems. The coordination mode of the complexes was defined using spectroscopic methods: electron paramagnetic resonance (EPR), ultraviolet-visible (UV-Vis), circular dichroism (CD), and infrared (IR). Results of the equilibrium studies have provided evidence for the formation of dimers with copper(II) ions and monomers with cobalt(II) and nickel(II) ions.

## 1. Introduction

Metal ions, which are found in living organisms, occur at the active sites of numerous enzymes and play a key role in them. Their complexes are interesting as model compounds of active sites of biological enzymes [1,2,3,4,5,6]. The divalent copper, cobalt, and nickel ions are examples of such metal ions. They play vital roles in transport of oxygen, enzyme function, cell growth, numerous enzymatic reactions, and the functioning of the central nervous system [7,8,9,10]. The biological role of copper(II) ions is well known. It affects the activity of enzymes such as Cu/Zn-superoxide dismutase, cytochrome oxidase, and ceruloplasmin, which are important for defense against free radicals, cell respiration, and melanin synthesis [11]. It has been proven that the accumulation of copper(II) ions can be toxic and cause serious disease (e.g., Huntington’s, Alzheimer’s, Parkinson’s, Wilson’s, and Menkes diseases) [12,13,14]. Cobalt(II) ion is an essential compound for the synthesis of vitamin B_12_ [15]. Classes of enzymes depending on vitamin B_12_ (e.g., isomerases, methyltransferases, reductive dehalogenases) participate in the reactions of DNA synthesis, fatty acid synthesis and production of energy [16]. Enzymes containing nickel(II) ions such as urease, hydrogenase, methyl-CoM reductase, and CO dehydrogenase/acetylCoA synthase (CODH/ACS) play a crucial role in the carbon cycle [17,18].

Tartaric acid (Tar) and malic acid (Mal) are four-carbon dicarboxylic species that play an essential biological role. Malic acid has one hydroxyl group, while tartaric acid has two hydroxyl groups. (*R*,*R*)-tartaric acid and its derivatives are useful in organic pharmaceutical chemistry for drug preparation (granules, effervescent, and others) and as an acidity regulator or stabilizing substance [19,20,21,22,23]. The antioxidant properties and coordination ability of malic acid are also used in medicine and pharmacology [24]. Optical properties naturally occur in tartaric and malic acids, their salts and derivatives play an important role in pharmaceutical and organic chemistry [25] not only due to their historical significance [26] but also to their present applications, [19,20,21,22,23,27]. Knowing conformational preferences [23,28,29,30] of these chiral ligands can support and broaden the knowledge of the role of their behavior in biological and chemical systems both in crystals [31] and in solutions [32]. Moreover, these acids and their ability to complexation with biologically active d-electron metal ions may be useful to design new generation drugs. These types of complexes can be suitable in chelation therapy for metal ions such as copper(II) ions [33,34,35]. The presence of an asymmetric hydroxyl group relative to the carboxyl group offers the opportunity to create different ion-dipol orientations in complex formation with metal ions: mononuclear and polynuclear species [24,36].

The ability of α-hydroxy acids to form complexes in more complicated complex systems in which there are other bioligands, e.g., proteins fragments, indicates a potential application of this type of complex in medicine. Attempts to search for new anticancer drugs with complexes with alfa hydroxy acids still give hope, especially in therapy with the parallel use of antimetabolites together with other chemotherapeutic compounds. The properties of new complex compounds, including those with tartaric and malic acids, may allow them to be used, for example, in cosmetology, medical diagnostics, and immunological tests.

Continuing our systematic studies on hydroxy acids, we present here the potentiometric and spectroscopic studies of complex formation in the systems of L-tartaric acid or L-malic acid with copper(II), cobalt(II), and nickel(II) ions. The effect of concentrations of α-hydroxy acid was also investigated.

## 2. Results and Discussion

### 2.1. Literature Comparison

The results described in the paper were different from those described in the literature [37,38,39]. Differences result of the different research conditions in the literature e.g., ionic strength (0.1 M NaNO_3_), temperature (25 °C), molar ratio (1:1 to 1:4), ligand concentration (0.001 M to 0.006 M). In our work, we observed small differences in the values of the overall stability constants (log*β*) and other complex forms. Furthermore, the formation of complex forms in binary systems was determined at a pH of about 7.0. In previous works, chemical models included only the formation of monomeric forms ML and ML_2_. Results of literature potentiometric data of copper(II), nickel(II), cobalt(II) ions in systems with L-tartaric and L-malic acids was present in Table 1.

In addition, we performed more repeats of the potentiometric measurements of each system that contained more titration points used in the calculation (compared to the maximum of 120 points in the literature). Moreover, we used different spectroscopic methods (EPR and CD) to confirm the formation of new detected complexes, e.g. dimers, which will be essential in future studies.

### 2.2. Binary Systems of Copper(II) Ion/α-Hydroxy Acid

In the tested pH range 2.5–11.0, the carboxyl groups of malic and tartaric acids are deprotonated and can be potential coordination centers. The values of the protonation constants of the hydroxyl groups for the α-hydroxy acids were determined by ^13^C NMR spectroscopy and is from about 11.6 to about 14.0 [40,41]. Such a high value causes hydroxyl groups to be ignored in studies of complex formation in aqueous solutions.

Relatively low p*K_a_* values and protonation constants (Table 2) of the dissociation of carboxyl groups of the studied α-hydroxy acids indicate that the deprotonation of these groups occurred at low pH values. In biological systems, these compounds can occur in partly or fully deprotonated forms, which allow the formation of complexes with metal ions such as copper(II), cobalt(II), or nickel(II).

**Figure 1:**
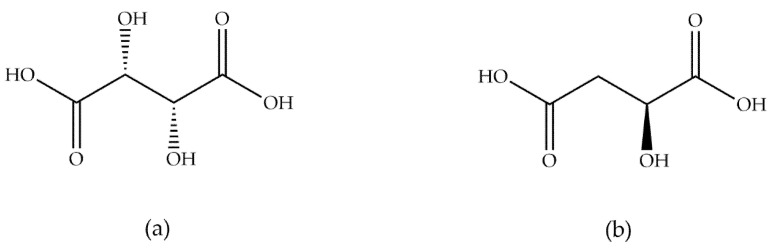
Formulae of studied ligands: (**a**) L-tartaric acid; (**b**) L-malic acid.

Analysis of the obtained p*K_a_* values for the studied acids confirmed that tartaric acid shows stronger acid properties than malic acid, which contains only one hydroxyl group in the structure. Structural formulae of the studied acids are displayed in Figure 1.

Computer analysis of the potentiometric data of the binary systems of L-tartaric or L-malic acids has shown the formation of complexes of similar type with copper(II) ions. For these systems, the formations of both monomeric and dimeric forms were found. The presence of two acid molecules in the internal coordination sphere was observed only in systems with an excess amount of ligands. The values of the overall stability constants (log*β*) and the equilibrium constants of complex formation (log*K_e_*) are presented in Table 1. Equilibrium constants (log*K_e_*) of binary complexes were determined based on the proposed reaction of their formation oM + pL + qH ⇆ M_o_L_p_H_q_ (for simplicity, the ion charges in the potentiometric description of the complexes were omitted) (Table 2). The results of the presented research will be used in more complicated systems that can be used in medical diagnostics, which is important to maintain exactly the same conditions. The correctness of the selected model was confirmed by the coincidence of experimental and theoretical curves, as well as relatively low values of the standard deviation of log*β*.

**Figure 2:**
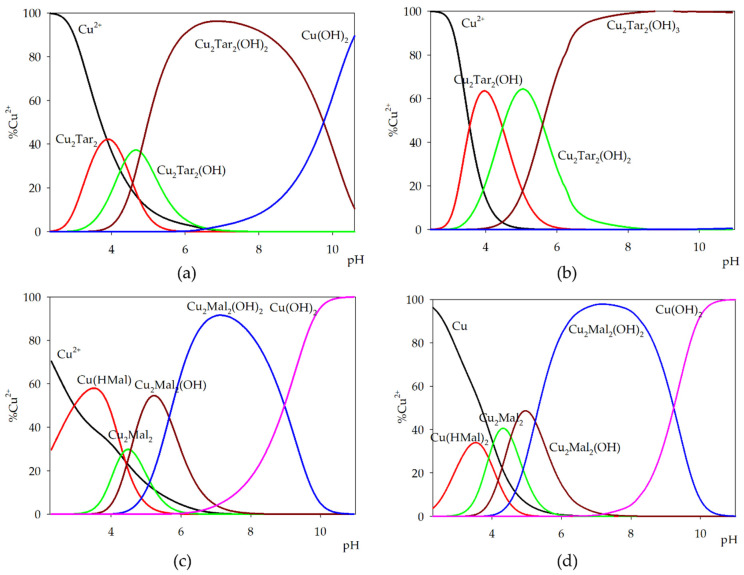
Distribution diagram for the binary systems: (**a**) Cu(II)/tartaric acid (1:1 ratio); (**b**) Cu(II)/tartaric acid (1:2 ratio); (**c**) Cu(II)/malic acid (1:1 ratio); and (**d**) Cu(II)/malic acid (1:2 ratio).

In equimolar systems, the complexation process begins with the formation of the M(HL) species in the system with L-malic acid (Figure 2c) and the dimeric form M_2_L_2_ in the reaction with L-tartaric (Figure 2a). In the Cu(II)/Mal system, the Cu(HMal) complex binds about 60% of the metal ions and is the dominant form at pH around 3.5, while in the system with tartaric acid, this form is not detectable. At the pH of the dominance of Cu(HMal), the dimeric complex Cu_2_Mal_2_ is formed, while Cu_2_Tar_2_ is the dominant form in the system at pH 3.9. Compared to the system with tartaric acid, the hydroxyl complex Cu_2_Mal_2_(OH) is the dominant species in the system. At neutral pH, the dominant complex is Cu_2_L_2_(OH)_2_, binding almost 100% of the metal ions. The presence of complexes in the system disappears at pH 11.0, when copper(II) hydroxide forms.

In the system with L-tartaric acid, only the dimeric forms were observed. The participation of two molecules in the coordination sphere of the central ion was recorded in the monomeric complex of copper(II) ions with L-malic acid Cu(HMal)_2_. The formation of protonated complexes was observed only in systems containing an excess of malic acid (Figure 2d). This complex formed at low pH values binding a maximum of 30% of copper(II) ions. The formation of copper(II) ions complexes with two fully deprotonated malic acid molecules was observed at pH about 3.0. At the pH of his maximum concentration, the dimer Cu_2_Mal_2_ binds about 40% of the metal ions presented in the system. At pH above 4.0, hydroxy complexes Cu_2_Mal_2_(OH) and Cu_2_Mal_2_(OH)_2_ were observed. Nearly 100% of copper(II) ions are related in the form of a dihydroxy complex. The increased amount of L-tartaric acid, compared to metal ions, resulted in the formation of a similar set of complexes as in the equimolar system excluding the formation of Cu_2_Tar_2_ (Figure 2b). As follows, the distribution that formed hydroxy complexes Cu_2_Tar_2_(OH) and Cu_2_Tar_2_(OH)_2_ at the pH of their dominance bound about 60% of copper(II) ions. Additionally, along with the increase in pH value, Cu_2_Tar_2_(OH)_3_ was observed as a dominant form, where 100% of the metal ions were involved in the formation of the complex in the pH range of 8.0–11.0.

**Figure 3:**
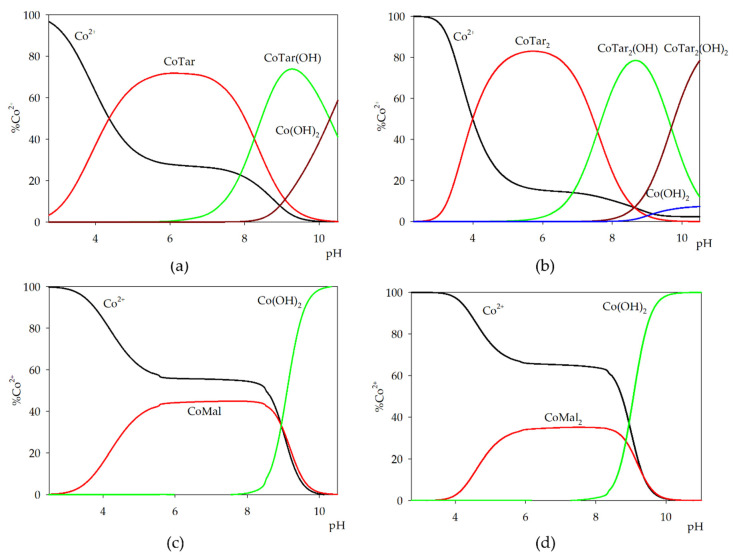
Distribution diagram for the binary systems: (**a**) Co(II)/tartaric acid (1:1 ratio); (**b**) Co(II)/tartaric acid (1:2 ratio); (**c**) Co(II)/malic acid (1:1 ratio); and (**d**) Co(II)/malic acid (1:2 ratio).

**Figure 4:**
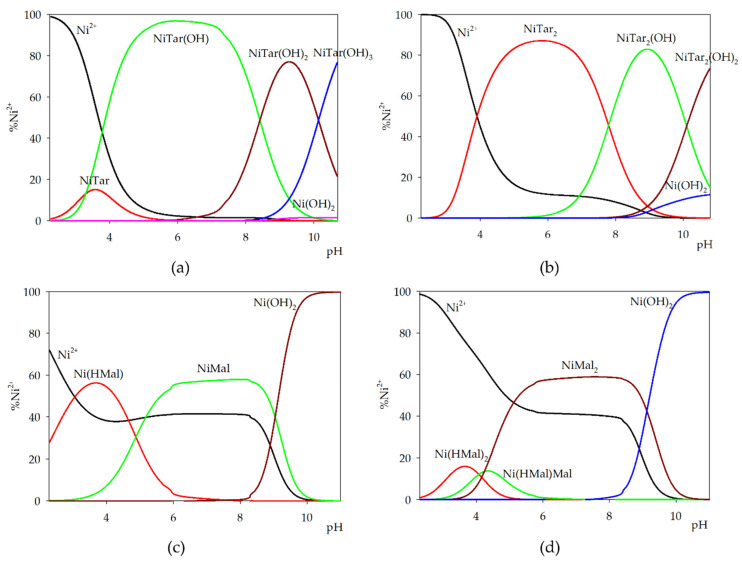
Distribution diagram for the binary systems: (**a**) Ni(II)/tartaric acid (1:1 ratio); (**b**) Ni(II)/tartaric acid (1:2 ratio); (**c**) Ni(II)/malic acid (1:1 ratio); and (**d**) Ni(II)/malic acid (1:2 ratio).

In systems with cobalt(II) and nickel(II) ions, only monomeric complexes were observed (Figure 3 and Figure 4). In systems with cobalt(II) ions with both hydroxy acids (molar ratio is 1:1), complexation began with the formation of the ML complex. Moreover, in the system with malic acid, this was the only one observed forming and binding about 45% of metal ions, as shown in Figure 3. In the system with tartaric acid at pH = 6.0, 60% of free metal ions were involved in complex CoTar. At the pH of the dominance of the CoTar form, the hydroxy complex CoTar(OH) started to form and was dominant above the pH near to 8.0.

In systems with cobalt(II) ions and an excess of α-hydroxy acid, two molecules of acid were involved in coordination. In the system with malic acid, only one form occurred, CoMal_2_ (pH range 4.0–10.0 and binding about 35% of the metal ions). In the tartaric acid system, the first CoTar_2_ complex formed and involved about 80% of the free metal ions introduced into the system. At the pH of the dominant CoTar_2_ complex, the formation of CoTar_2_(OH) was observed. In contrast to the equimolar system, a dihydroxy complex of cobalt ions was detected (Figure 3b).

L-Tartaric acid with nickel(II) ions in an equimolar system formed at pH 2.5 NiTar complex and a hydroxy complex Ni(Tar)(OH) (Figure 4a). The hydroxy complex was already dominant in the pH range of about 3.0 to 8.0 and bound nearly 100% of metal ions in the pH range 5.0–7.0. At the dominance of this form, di-hydroxy complex started to form and bound about 80% of nickel(II) ions. In the system with malic acid at low pH values, the protonated form of the complex was observed (Figure 4c). Increasing of pH value led to full deprotonation of L-Malic acid and NiMal complex was formed. Both forms involved nearly 60% of the metal ions introduced to the system. Moreover, free metal ions were observed almost across the entire studied pH range, such as in all systems with cobalt(II) ions.

In the systems with double excess of ligands, two molecules of acid were observed in the internal coordination sphere. More than 80% of the metal ions were involved in the formation of NiTar_2_ complex. At higher pH values, the hydroxy complexes formed were: NiTar_2_(OH) and NiTar_2_(OH)_2_ (Figure 4b). The computer analysis of potentiometric data collected for the system with a double excess of malic acid showed the formation of three types of complexes. From pH 2.5, the presence of Ni(HMal)_2_ and Ni(HMal)Mal was detected and 20% of the metal ions introduced into the system were involved in coordination (Figure 4d). Fully deprotonated molecules of α-hydroxy acid appeared at pH about 4.0 and NiMal_2_ complex started forming (dominant in the pH range 4.5–9.0).

In binary systems of α-hydroxy acid with d-electron metal ions, higher values of overall stability constants (log*β*) of analogous complex forms for tartaric acid compared to malic acid were observed. In addition, the differences in the acidic properties of these compounds have an influence on the types of complexes formed in the systems. The degree of their deprotonation is especially noticeable at low pH values, where the formation of protonated forms is observed for systems with malic acid. Additionally, the maximum concentration of analogous complexes was shifted to a higher pH value for malic acid and at pH 11.0 only copper(II) hydroxide was observed where with tartaric acid, hydroxy complexes are dominant. The analysis of the obtained results indicates a higher affinity of metal(II) ions in order: copper(II) ions > nickel(II) ions > cobalt(II) ions for the coordination with oxygen atoms of the carboxyl groups of the studied ligands. Similar to our previous study with citric acid [42], it was observed that copper(II) ions in the equimolar ratio and with double excess of ligands showing a tendency to form dimeric complexes. In contrast to these ions, cobalt(II) and nickel(II) ions, in systems with excess amounts of studied acids, formed complex compounds in which two ligand molecules were located in the inner coordination sphere of the central atom.

### 2.3. Spectroscopic Study

#### 2.3.1. UV-Vis Spectroscopy and EPR Spectra

The formation of complex compounds in systems containing d-electron metal ions was confirmed using spectroscopic methods. By selecting the optimal pH values, ensuring the highest possible percentage of the form of a given complex (based on distribution diagrams), UV-Vis measurements were performed for the studied systems. The spectral data obtained for the complexes are presented in Table 3.

**Figure 5:**
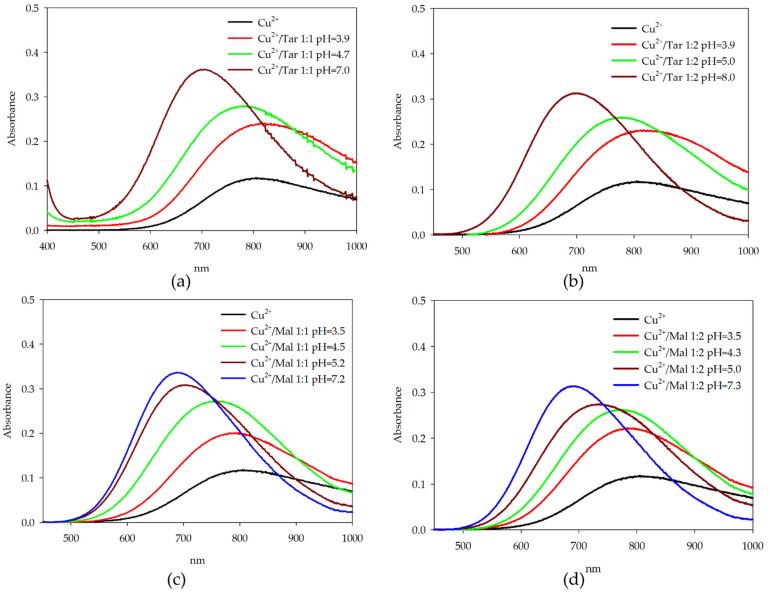
UV-Vis absorption spectra of: (**a**) Cu(II)/Tar 1:1; (**b**) Cu(II)/Tar 1:2; (**c**) Cu(II)/Mal 1:1; and (**d**) Cu(II)/Mal 1:2.

In systems with copper(II) ions, the shift of the absorbance toward lower wavelengths indicates a change in the internal coordination sphere of the metal ions (Figure 5). By the attachment of an additional oxygen atom of the α-hydroxy acid molecule to copper(II) ions, changes in spectral parameters were observed. Differences in the obtained spectral data for analogous complex forms followed from differences in the percentage of copper ions that are involved in the formation of a complex compound.

**Figure 6:**
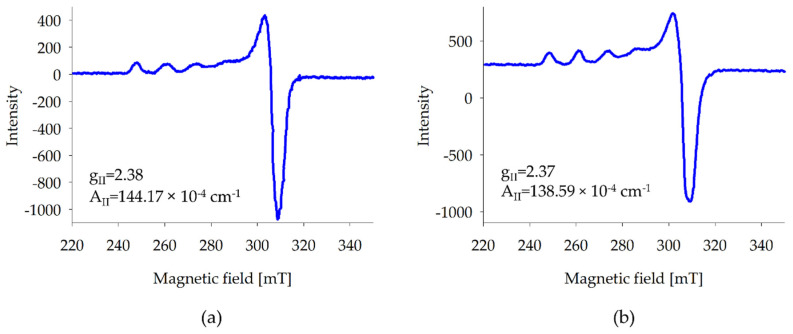
EPR Spectra of: (**a**) Cu(HMal) and (**b**) Cu(HMal)_2_.

The occurrence of both dimeric and monomeric forms in the copper(II) ion systems was confirmed by EPR measurements. At low pH values, monomeric forms are dominant, and characteristic spectra for copper(II) ions were observed (Figure 6). For the protonated complex compound Cu(HMal), the spectral parameters indicate the involvement of one oxygen atom from partially deprotonated L-malic acid molecule. In the formation of Cu(HMal)_2_, the EPR parameters: g_II_ = 2.37 and A_II_: 138.59 × 10^−4^ cm^−1^, imply the coordination two oxygen atoms. The extinction of the EPR spectrum signal observed for at pH value of dominance dimeric forms confirms the presence of complexes containing two copper(II) ions in the molecule (the characteristic signal for copper(II) ions disappears resulting from spin coupling). EPR silence informs that the dinuclear species are an antiferromagnetic exchange interaction and that two cations have coplanarity of their d_xy_ orbitals.

**Figure 7:**
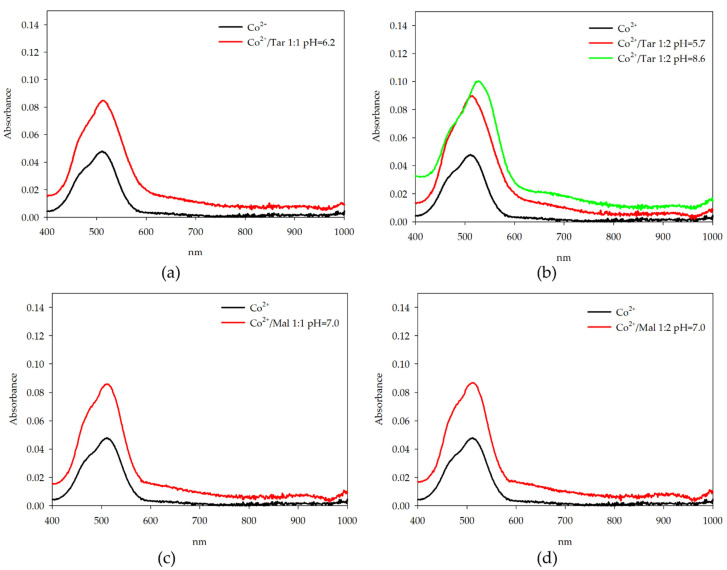
UV-Vis absorption spectra of: (**a**) Co(II)/Tar 1:1; (**b**) Co(II)/Tar 1:2; (**c**) Co(II)/Mal 1:1; and (**d**) Co(II)/Mal 1:2.

In systems containing cobalt(II) ions in complexes with fully deprotonated acids, the d-d parameters suggest similar coordination in CoL and CoL_2_ forms. Small shifts of the wavelength at maximum absorption for CoTar_2_(OH) to longer wavelengths were observed (Figure 7). In pH values in which the species CoTar(OH) and CoTar_2_(OH)_2_ are dominant, it was impossible to record UV-Vis spectra because of precipitate formation.

**Figure 8:**
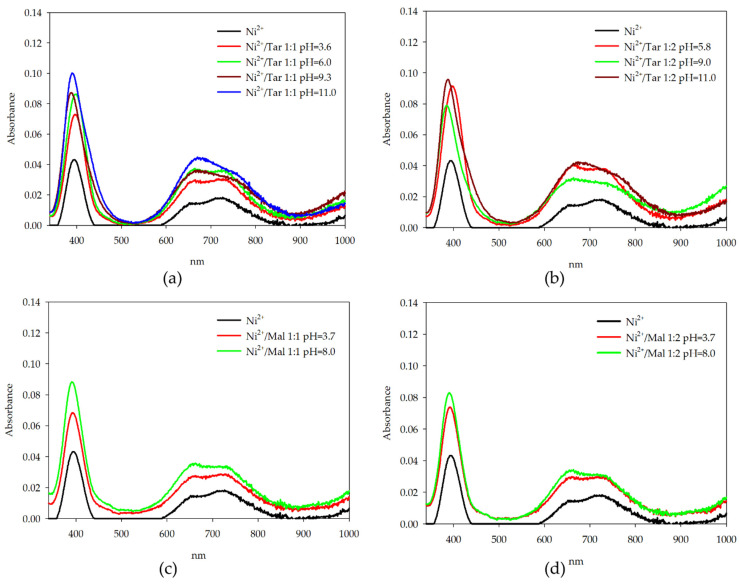
UV-Vis absorption spectra of: (**a**) Ni(II)/Tar 1:1; (**b**) Ni(II)/Tar 1:2; (**c**) Ni(II)/Mal 1:1; and (**d**) Ni(II)/Mal 1:2.

For the complexes of nickel(II) ions with L-tartaric acid and L-malic acid, small blue-shift is noted (Figure 8). The molar absorption coefficient for the maximum absorption wavelength increased along with increasing pH values. Based on the spectra obtained for systems with nickel ions, the largest changes were observed in wavelength range 600–800 nm. Overall, these results suggest a change in the internal coordination sphere of metal ion with an increase in pH [43].

#### 2.3.2. IR Spectroscopy

**Figure 9:**
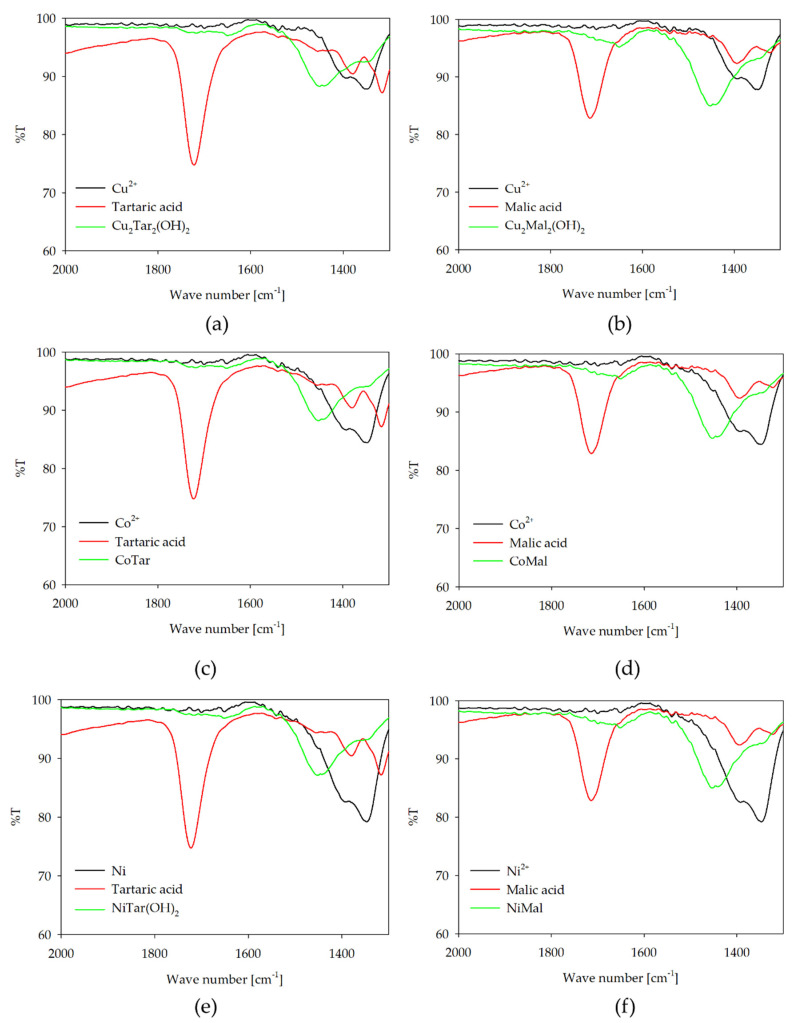
IR spectra of: (**a**) Cu(II)/tartaric acid; (**b**) Cu(II)/malic acid; (**c**) Co(II)/tartaric acid; (**d**) Co(II)/malic acid; (**e**) Ni(II)/tartaric acid; and (**f**) Ni(II)/malic acid.

The involvement of carboxyl groups of L-tartaric and L-malic acids in the coordination of complex compounds with the studied d-electron metal ions was confirmed using IR spectroscopy. The IR spectrum was recorded at the dominant of the pH of complex compounds. Comparative spectra were made for the tested ligands and metal ions under the same pH conditions. In all systems studied, the characteristic asymmetric stretching vibration of the C=O bonds of the carboxyl group (ν_asC=O_ near 1700 cm^−1^) disappeared for the spectra of the complex forms, compared to the spectrum of the free ligands. Interpretation of the spectroscopic information shows the involvement of carboxyl groups in complex formation with metal ions. Furthermore, in the range of symmetric stretching, vibration of the C–O bonds (ν_asC–O_ 1450–1350 cm^−1^) are observed. The shifts observed in this range, compared to the spectra of free ligands and solutions of metal salts, confirm the formation of coordination bonds between metal ions and donor groups of alfa hydroxy acids. The participation of carboxyl groups in the formation of the complex forms in the equimolar systems is presented in Figure 9.

#### 2.3.3. CD Spectroscopy

The CD and corresponding UV spectra were recorded for all systems with (*R*,*R*)-tartaric and (*S*)-malic acid in water in the range of 185–350 nm at a concentration of 0.001 M. Using a sample cell with 0.5 mm optical pathlength allowed measurements of all samples at relatively high concentration, which allowed a direct comparison with the results of titration measurements. In two cases of samples that were not fully soluble, CD spectra were scaled to UV spectra (values in italics in Table 3). All series of samples were measured at pH, determined on the basis of the results obtained by computer analysis of potentiometric data.

Tartaric and malic acids do not have aromatic groups incorporated in their structures, which is commonly used in the analysis of conformational preferences of molecules by CD spectroscopy. The analysis of their conformation, and thus the interactions with ions were conducted on the basis of observations of Cotton effects derived from the n-π* electron transitions of carboxylic groups in these molecules. This limited the range of observations between 185 and 250 nm.

In this analysis, the natural conformational preferences of tartaric and malic acids must be considered. It is known that in protonated and deprotonated forms, optically active tartaric acid mainly adopts a trans conformation of its carbon backbone, but as a result of ionization and rearrangement of intramolecular hydrogen bonds, its CD spectra change its shape at different pH. (*R*,*R*)-Tartaric acid (L-tartaric acid) in aqueous solution exhibits a single negative Cotton effect Δε = −4 around 210–215 nm and its simple (Na, K, Li) divalent salts show two negative Cotton effects Δε = −4 at 193 nm and −2.5 at around 211 nm. In the case of non-racemic malic acid, the change in its CD spectra also depends on the pH of the solution. (*S*)-malic acid (L-malic acid), used in experiments in aqueous solutions, exhibits a single positive Cotton effect Δε = 1.3 at 211 nm and its simple (Na, K, Li) divalent salts show positive Cotton effect Δε = 3.8 at 208 nm. All the above-mentioned changes were considered during the analysis process.

In the CD spectrum of a solution of copper and tartaric acid (in a 1:1 ratio) at pH 3.5, a negative Cotton effect Δε = −2.77 at 213 nm can be observed, which indicates that most of the acid remains uncomplexed and adopts the trans conformation. At pH 4.7 (for Cu(II):Tar ratio 1:1) and 4.2 (for Cu(II):Tar ratio 1:2) in both samples, positive Cotton effects in CD spectra can be observed at about 235 nm, indicating the adoption of a gauche conformation by the molecule. The Cotton effect at 207 nm present in CD spectrum for a sample with 1:2 ratio indicates that the second equivalent of the tartaric acid molecules remains in the solution in the trans conformation. Raising the pH to the ones in which the Cu_2_Tar_2_OH form dominates in the solutions does not change the pattern of the Cotton effects in CD spectra, which means that the conformation of the carbon skeleton of tartaric acid does not change significantly.

**Figure 10:**
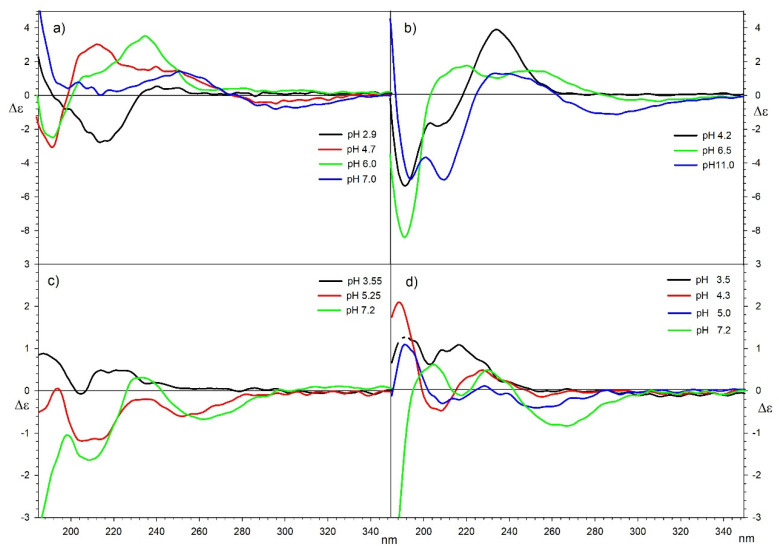
CD spectra of complexes of copper and (*R*,*R*)-tartaric acid in the ratios of 1:1 (**a**) and 1:2 (**b**), and (*S*)-malic acid in the ratios of 1:1 (**c**) and 1:2 (**d**).

The decrease in Cotton effect in the long-wavelength region of pH in which Cu_2_Tar_2_(OH)_2_ form dominates, indicates a conformational change in which certain tartrates change its structure to a trans conformation. This is most likely due to the high concentration of negative charge in the resulting structures (Figure 10a,b).

The CD spectra of copper-malic acid complexes for a pH close to 3.5 indicate partial complexation of the central atom by the acid. The positive Cotton effect at about 210 nm suggests that acid remained uncomplexed in the trans conformation. This effect disappears for measurements performed at pH 4.3 (1:2 ratio), 5.0 (1:2 ratio) and 5.25 (1:1 ratio), which is associated with the formation of a complex in which malic acid changes the conformation from trans to gauche. Possibly due to the larger volume of binary complexes of copper, unlike in cobalt and nickel complexes, the CD spectra do not change significantly when the hydroxyl groups are attached to the complex at pH 7.2 (Figure 10c,d).

Both measurements of circular dichroism spectra of nickel-tartaric acid complexes in the ratio 1:1 and 1:2 show a similar course of complexation depending on the pH. For an equimolar nickel-tartaric acid complex measured at a pH of 3.7, the CD spectrum indicates the presence of a predominantly non-ionized trans acid. Measurements at the pH of about 6 indicate an increase in ionization while maintaining the trans conformation. Positive Cotton effect at ca 218 nm of Δε = 2.68 and Δε = 3.74 for 1:1 and 1:2 complexes at pH 9.25 and 9.0 respectively, are connected with conformation changes of ionized tartaric acid. This effect disappears for the measurements of the complexes at a pH of 11.0. This is due to the second hydroxylic group complexing the central nickel atom, thus changing the conformation of the acid. In this case, the return to the trans conformation can also be caused by the repulsion of the negative charges of the hydroxyl groups and of the tartaric acid anions.

The CD spectra of the nickel-malic acid 1:1 and 1:2 complexes measured at a pH of 3.7 (Δε = 0.62 at 215nm, Δε = 0.65 at 210 nm for 1:1 ratio and Δε = 1.69 at 210 nm and Δε = 0.85 at 194nm for 1:2 ratio) indicate that, at this pH, most of the malic acid remains unbound in solution and adopts a trans conformation. Comparison of the CD spectra of nickel complexes at pH 8.0, with one and two acid equivalents exhibits a decrease of the positive Cotton effect at 210 nm observed in both cases and indicates the formation of complexes with one acid molecule for an equimolar mixture and two acid molecules for a mixture with double excess of malic acid.

Comparison of CD measurement results of the 1:1 and 1:2 cobalt to tartaric acid molar ratio implies that only one equivalent of acid participates in the coordination process while the other does not. The contribution of the second tartaric acid molecule to the CD spectrum is almost identical to the CD spectrum of its simple salts. The decrease in the intensity of the Cotton effects at the shortest wavelengths indicates that, as in the case of nickel complexes, the introduction of hydroxyl groups into the complexes involves a change in the conformation of tartaric acid. As a result of the low solubility of cobalt complexes of tartaric acid at higher pH, it was necessary to scale the measurement results to the UV spectrum.

CD spectra of malic acid complexes with cobalt exhibited the Cotton effects Δε = 0.68 (210 nm) and Δε = 1.25 (195 nm) for equimolar system and Δε = 2.23 (209 nm), Δε = 2.54 (195 nm) for 1:2 ratio and present relatively small decrease of Cotton effects in comparison to uncomplexed malic acid at corresponding pH. This indicates that a significant part of malic acid stays unbound in solution, which is in agreement with potentiometric studies.

The results of the circular dichroism measurements containing the Cotton effects maxima and the corresponding wavelengths are summarized in Table 4 for tartaric acid and Table 5 for malic acid.

## 3. Materials and Methods

### 3.1. Materials

L-tartaric acid (Tar) and L-malic acid (Mal) were obtained from Merck and used without further purification. Copper(II), cobalt(II), and nickel(II) nitrates from Sigma-Aldrich (Steinheim am Albuch Baden-Württemberg, Germany) were purified by recrystallization from water. In the first step, the complexometric methods were used for determination of the concentration of metal(II) ions in the solution, and next, were confirmed by inductively coupled plasma optical emission spectrometry (ICP OES) (Shimadzu, Kyoto, Japan). All solutions, which were used in the experiments, were prepared using demineralized carbonated-free water (conductivity 0.055 µs).

### 3.2. Equilibrium Study

Potentiometric titrations were conducted using a Titrando 905 Metrohm equipped with an autoburette with an i-electrode Metrohm 6.0280.300 (Metrohm AG, Herisau, Switzerland) calibrated in terms of concentration of hydrogen ions prior to each titration [44,45,46,47,48,49]. The pH meter indication (Metrohm AG, Herisau, Switzerland) was corrected by two standard buffer solutions of pH 4.002 and pH 9.225 before each series of measurements. Each potentiometric titration was conducted under constant and strictly defined conditions: constant ionic strength of 0.1 M (KNO_3_), temperature 20 ± 1 °C (titration dish placed in thermostatic bath set at this temperature), an atmosphere of neutral gas (helium—Ultra High Purity 5.0; (Linde Gaz, Krakow, Poland), pH range from 2.5 to 11.0, and titrant CO_2_-free NaOH (titrant addition step—0.006 mL). The measurements were conducted at the metal to ligand molar ratios 1:1 and 1:2 in which the concentration of metal ions was 0.001 M, and it was repeated 8 times. The protonation constants and stability constants of the complexes were determined using the HYPERQUAD 2008 program (Hyperquad Limited, Leeds, UK) by using 150–350 points from each titration. The calculations allowed determination of the model of complex formation in the studied systems. The correctness of the assumed model was verified by analyzing the standard deviations and the convergence of the experimental curve to that obtained from the model and was evaluated by the Hamilton test and the chi-square test [49,50,51]. The following equilibria oM + pL + qH ⇆ M_o_L_p_H_q_ stability constants were evaluated (where M = metal ion, L = ligand), and calculated using the following equation:

(1) $$\beta \;=\;\frac{M_{o}L_{p}H_{q}}{{\left[M\right]}^{o}{\left[L\right]}^{p}{\left[H\right]}^{q}}$$

The computer analysis of the potentiometric data included hydrolysis constants of metal ions (taken from our previous article [52]) and the ionic product for water was p*K*_w_ = 13.78 [44,46,47,48]. The distribution diagrams of particular forms were obtained using the HySS (Hyperquad Simulation and Speciation) program [44].

### 3.3. UV-Vis Spectroscopy

The UV-Vis absorption spectra were recorded at room temperature on Evolution 300 UV-Vis ThermoFisher Scientific (Thermo Electron Scientific Instruments LLC, Madison, WI, USA) (resolution 0.2 nm) equipment with a xenon lamp using a Plastibrand PMMA cell (Brand, Wertheim, Germany) with 1 cm path length. Measurements were performed in the wavelength range of 340–1000 nm. The concentration of metal ions was 0.01 M and the metal to ligand molar ratios were 1:1 and 1:2.

### 3.4. Infrared Spectroscopy

Samples for IR spectra were performed by dissolving the relevant species (tartaric acid, malic acid and metal ions) in D_2_O. Spectra were recorded on an FT-IR INVENIO R Spectrophotometer (Bruker, Bremen, Germany) and measured for free ligands and binary systems (metal to ligand molar ratios were 1:1 and 1:2). The metal concentration for the IR studies was 0.001 M. The pH values were adjusted by addition of NaOD or DCl. The pH values were corrected according to the formula pD = pH meter reading + 0.4 [49].

### 3.5. EPR Spectroscopy

The EPR spectra were conducted at −196 °C (temperature of liquid nitrogen), using glass capillary tubes (volume 130 µm^3^) and were recorded on an SE/X 2457 Radiopan spectrometer (Radiopan, Poznan, Poland). EPR studies were performed for copper(II) ion systems, in which the concentration of metal ions was 0.005 M in water:glycol mixture (3:1), and the metal:ligand ratios 1:1 and 1:2.

### 3.6. CD Spectroscopy

The spectra of circular dichroism were recorded on a Jasco J810 spectropolarimeter (Jasco, Tokyo, Japan) (resolution 1 nm) in nitrogen gas in the range from 185 to 350 nm at room temperature. The concentration of the relevant species (prepared in demineralized water) was the same as in the equilibrium study and the optical path length was 0.5 nm.

All spectra were recorded at ambient temperature, in the range of 185–350 nm in water solutions and were accumulated with 4 scans for tartaric acid complexes, and due to low signal to noise ratio, 10 scans for malic acid complexes. The measurements were performed in N_2_ atmosphere (flow 10 L/min) and optical pathlength was 0.5 mm. For well soluble samples, concentrations were of 1 × 10^−3^, and in the case of samples of non-sufficient solubility, the CD spectra were scaled to UV spectra.

## 4. Conclusions

Formation of binary complexes in binary systems of L-tartaric acid and L-malic acid with d-electron metal ions has been established. Different modes of coordination in the complexes were observed. Copper(II) ions tend to form dimers, which is confirmed by EPR spectra. Monomeric complexes were observed in systems with cobalt(II) and nickel(II) ions. In these ions, with double excess of ligands, two ligand molecules are located in the coordination sphere. Change of the inner coordination sphere was studied by spectroscopic methods. IR spectroscopy confirms the participation of carboxyl groups of studied α-hydroxy acids in formation of complex compounds. Shift of the absorbance toward lower wavelengths, the change of absorption, and the molar absorption indicates the attachment of an additional oxygen atom to metal ions. The results of this study suggest that L-tartaric acid shows a slightly higher tendency to form complexes with d-electron metal ions, compared to L-Malic acid. Overall, these results indicate that the higher affinity of metal(II) ions for coordination with the studied ligands was observed in order: copper(II) ions > nickel(II) ions > cobalt(II) ions. Moreover, these results will be used in more complicated systems and can be useful in medical diagnosis.

## Figures and Tables

**Table 1:** Literature data of the overall stability constants (log*β*) or equilibrium constants of formation (log*K_e_*) of the complexes formed in the binary system metal(II) ions/alfa-hydroxyacid.

	Species	L-Tartaric Acid (Tar)			L-Malic Acid (Mal)		
		log*β* [37]	log*β* [38]	log*K_e_* [39]	log*β* [37]	log*β* [38]	log*K_e_* [39]
H^+^	HL	2.96	2.96	-	3.40	5.26	-
	H_2_L	7.33	7.33	-	8.60	8.65	-
Cu^2+^	ML	6.96	6.91	3.90	6.80	6.75	4.01
	ML_2_	5.68	5.65	-	-	-	-
Co^2+^	ML	3.15	3.20	2.85	3.12	3.10	3.08
	ML_2_	2.37	2.40	-	-	-	-
Ni^2+^	ML	4.67	4.61	3.06	4.63	4.60	3.35
	ML_2_	3.38	3.35	-	-	-	-

**Table 2:** Protonation constants of α-hydroxy acids, the overall stability constants (log*β*) and equilibrium constants of formation (log*K_e_*) of the complexes formed in the binary system d-electron metal ions /alfa-hydroxy acid (standard deviations are given in parentheses).

	Species	L-Tartaric Acid (Tar)		L-Malic Acid (Mal)		Reaction
		log*β*	log*K_e_*	log*β*	log*K_e_*	
L	H_2_L	7.08(1)	2.91	7.92(1)	3.22	HL^−^ + H^+^ ⇆ H_2_L
	HL	4.17(1)	4.17	4.70(1)	4.70	L^2−^ + H^+^ ⇆ HL^−^
Cu^2+^	M(HL)_2_	-	-	15.29(3)	5.89	M^2+^ + 2HL ⇆ M(HL)_2_
	M(HL)	-	-	8.96(3)	4.26	M^2+^ + HL ⇆ MLH
	M_2_L_2_	12.63(7)	12.63	11.37(5)	11.37	2M^2+^ + 2L ⇆ M_2_L_2_
	M_2_L_2_(OH)	8.06(1)	9.20	6.80(5)	9.20	M_2_L_2_ + H_2_O ⇆ M_2_L_2_(OH) + H^+^
	M_2_L_2_(OH)_2_	3.41(2)	9.12	1.13(3)	8.10	M_2_L_2_(OH) + H_2_O ⇆ M_2_L_2_(OH)_2_ + H^+^
	M_2_L_2_(OH)_3_	−2.03(3)	8.33	-	-	M_2_L_2_(OH)_2_- + H_2_O ⇆ M_2_L_2_(OH)_3_ + H^+^
Co^2+^	ML	3.92(3)	3.92	3.06(2)	3.06	M^2+^ + L ⇆ ML
	ML(OH)	−4.33(3)	5.52	-	-	ML + H_2_O ⇆ ML(OH) + H^+^
	ML_2_	7.92(2)	4.00	5.56(4)	2.50	ML + L ⇆ ML_2_
	ML_2_(OH)	0.07(2)	5.92	-	-	ML_2_ + H_2_O ⇆ ML_2_(OH) + H^+^
	ML_2_(OH)_2_	−10.15(3)	3.55	-	-	ML_2_(OH) + H_2_O ⇆ ML_2_(OH)_2_ + H^+^
Ni^2+^	M(HL)_2_	-	-	14.72(6)	5.32	M^2+^ + 2HL ⇆ M(HL)_2_
	M(HL)(L)	-	-	10.64(8)	5.94	M^2+^ + HL + L ⇆ M(HL)(L)
	M(HL)	-	-	8.40(5)	3.70	M^2+^ + HL ⇆ MLH
	ML	4.10(4)	4.10	3.52(4)	3.52	M^2+^ + L ⇆ ML
	ML(OH)	0.79(1)	10.46	-	-	ML + H_2_O ⇆ ML(OH) + H^+^
	ML(OH)_2_	−7.91(2)	5.07	-	-	ML(OH) + H_2_O ⇆ ML(OH)_2_ + H^+^
	ML(OH)_3_	−17.74(2)	3.94	-	-	ML(OH)_2_+ H_2_O ⇆ ML (OH)_3_ + H^+^
	ML_2_	8.14(1)	4.04	6.40(1)	2.88	ML + L ⇆ ML_2_
	ML_2_(OH)	0.34(2)	5.97	-	-	ML_2_+ H_2_O ⇆ ML_2_(OH) + H^+^
	ML_2_(OH)_2_	−9.75(2)	3.68	-	-	ML_2_(OH) + H_2_O ⇆ ML_2_(OH)_2_ + H^+^

Hydrolytic constants considered log*β*_Cu(OH)2_ = −13.13, log*β*_Co(OH)2_ = −17.89 and log*β*_Ni(OH)2_ = −17.79.

**Table 3:** Spectral parameters of binary complexes formed in systems of d-electron metal ions with L-tartaric acid or L-malic acid.

		Species	L-Tartaric Acid (Tar)			L-Malic Acid (Mal)		
			pH	λ_max_ (nm)	ε(M^−1^cm^−1^)	pH	λ_max_ (nm)	ε(M^−1^cm^−1^)
**Ratio 1:1**	Cu^2+^	M(HL)				3.5	790.0	29
		M_2_L_2_	3.9	820.0	27	4.5	757.0	31
		M_2_L_2_(OH)	4.7	780.0	32	5.2	704.0	36
		M_2_L_2_(OH)_2_	7.0	705.0	44	7.2	688.0	40
	Co^2+^	ML	6.2	511.0	9	7.0	512	9
		ML(OH)	9.3	---	---			
	Ni^2+^	M(HL)				3.7	391.0	8
		ML	3.6	396.0	8	8.0	391.0	10
		ML(OH)	6.0	399.0	10			
		ML(OH)_2_	9.3	390.0	11			
		ML(OH)_3_	11.0	390.0	13			
**Ratio 1:2**	Cu^2+^	M(HL)_2_				3.5	788.0	25
		M_2_L_2_				4.3	769.0	31
		M_2_L_2_(OH)	3.9	817.0	27	5.0	733.0	33
		M_2_L_2_(OH)_2_	5.0	780.0	32	7.3	690.0	40
		M_2_L_2_(OH)_3_	8.0	700.0	40			
	Co^2+^	ML_2_	5.7	515.0	10	7.0	512	10
		ML_2_(OH)	8.6	527.0	12			
		ML_2_(OH)_2_	11.0	---	---			
	Ni^2+^	M(HL)_2_				3.7	391.0	9
		M(HL)(L)				---	---	---
		ML_2_	5.8	397.0	12	8.0	391.0	11
		ML_2_(OH)	9.0	390.0	10			
		ML_2_(OH)_2_	11.0	388.0	14			

**Table 4:** Δε values for all Cu/Co/Ni/Tar systems at different pH values (numbers in italic represent spectra scaled to UV due to insufficient solubility).

Cu(II)/Tar	Cu(II)/2Tar	Co(II)/Tar	Co(II)/2Tar	Ni(II)/Tar	Ni(II)/2Tar
pH = 2.9		pH = 6.25	pH = 5.75	pH = 3.7	
Δε (nm)		Δε (nm)	Δε (nm)	Δε (nm)	
0.55 (240)		−1.66 (210)	−4.19 (210)	−3.94 (213)	
−2.77 (213)		−3.47 (193)	−7.64 (194)		
pH = 4.7	pH = 4.2	pH = 9.25	pH = 8.6	pH = 6.0	pH = 5.8
Δε (nm)	Δε (nm)	Δε (nm)	Δε (nm)	Δε (nm)	Δε (nm)
−0.44 (297)	3.9 (234)	−1.69 (213)	−3.80 (210)	−1.32 (210)	−3.73 (210)
1.73 (240)	−1.82 (207)	−2.04 (193)	−3.85 (207)	−3.25 (194)	−7.31 (193)
3.05 (213)	−5.35 (192)		−7.63 (193)		
−3.05 (192)					
pH = 6.0	pH = 6.5		pH = 11.0	pH = 9.25	pH = 9.0
Δε (nm)	Δε (nm)		Δε (nm)	Δε (nm)	Δε (nm)
3.51 (235)	1.51 (250)		−3.78 (211)	2.68 (218)	3.74 (219)
−2.49 (193)	1.80 (221)		−3.81 (208)	−3.69 (194)	−7.76 (193)
	−8.35 (192)		−6.14 (193)		
pH = 7.0	pH = 11.0			pH = 11.0	pH = 11.0
Δε (nm)	Δε (nm)			Δε (nm)	Δε (nm)
−0.8 (295)	−1.08 (286)			0.67 (225)	0.76 (229)
1.41 (251)	1.30 (233)			−0.59 (211)	−3.07 (210)
0.77 (204)	−4.99 (210)			−3.08 (194)	−7.35 (193)
	−4.97 (195)				

**Table 5:** Δε values for all Cu/Co/Ni/Mal systems at different pH values.

Cu(II)/Mal	Cu(II)/2Mal	Co(II)/Mal	Co(II)/2Mal	Ni(II)/Mal	Ni(II)/2Mal
pH = 3.55	pH = 3.5	pH = 7.0	pH = 7.0	pH = 3.7	pH = 3.7
Δε (nm)	Δε (nm)	Δε (nm)	Δε (nm)	Δε (nm)	Δε (nm)
0.48 (224)	1.09 (216)	0.68 (210)	2.23 (209)	0.62 (215)	1.69 (210)
0.48 (214)	0.96 (208)	1.25 (195)	2.54 (195)	0.65 (210)	0.85 (194)
0.88 (187)	1.26 (191)				
	pH = 4.3			pH = 8.0	pH = 8.0
	Δε (nm)			Δε (nm)	Δε (nm)
	−0.14 (255)			−1.75 (202)	1.11 (218)
	0.48 (228)				−0.22 (202)
	−0.48 (208)				4.35 (185)
	2.09 (189)				
pH = 5.25	pH = 5.0				
Δε (nm)	Δε (nm)				
−0.60 (252)	−0.40 (252)				
−1.15 (213)	0.15 (228)				
−1.18 (205)	−0.30 (209)				
	1.1 (191)				
pH = 7.2	pH = 7.2				
Δε (nm)	Δε (nm)				
−0.68 (262)	−0.83 (267)				
0.31 (233)	0.50 (230)				
−1.64 (208)	−0.10 (217)				
	0.63 (204)				

## Data Availability

The data presented in this study are not available from the authors.

## Sample Availability

Samples of the compounds are not available from the authors.

## Abbreviations

M	Metal
L	Ligand
Tar	Tartaric acid
Mal	Malic acid
